# Interactions of the apolipoprotein C-III 3238C>G polymorphism and alcohol consumption on serum triglyceride levels

**DOI:** 10.1186/1476-511X-9-86

**Published:** 2010-08-17

**Authors:** Yin Ruixing, Li Yiyang, Li Meng, Li Kela, Long Xingjiang, Zhang Lin, Liu Wanying, Wu Jinzhen, Yang Dezhai, Lin Weixiong

**Affiliations:** 1 Department of Cardiology, Institute of Cardiovascular Diseases, the First Affiliated Hospital, 22 Shuangyong Road, Nanning 530021, Guangxi, People's Republic of China; 2 Department of Molecular Biology, Medical Scientific Research Center, 22 Shuangyong Road, Nanning 530021, Guangxi, People's Republic of China

## Abstract

**Background:**

Both apolipoprotein (Apo) C-III gene polymorphism and alcohol consumption have been associated with increased serum triglyceride (TG) levels, but their interactions on serum TG levels are not well known. The present study was undertaken to detect the interactions of the ApoC-III 3238C>G (rs5128) polymorphism and alcohol consumption on serum TG levels.

**Methods:**

A total of 516 unrelated nondrinkers and 514 drinkers aged 15-89 were randomly selected from our previous stratified randomized cluster samples. Genotyping of the ApoC-III 3238C>G was performed by polymerase chain reaction and restriction fragment length polymorphism combined with gel electrophoresis, and then confirmed by direct sequencing. Interactions of the ApoC-III 3238C>G genotype and alcohol consumption was assessed by using a cross-product term between genotypes and the aforementioned factor.

**Results:**

Serum total cholesterol (TC), TG, high-density lipoprotein cholesterol (HDL-C), ApoA-I and ApoB levels were higher in drinkers than in nondrinkers (*P *< 0.05-0.001). There was no significant difference in the genotypic and allelic frequencies between the two groups. Serum TG levels in nondrinkers were higher in CG genotype than in CC genotype (*P *< 0.01). Serum TC, TG, low-density lipoprotein cholesterol (LDL-C) and ApoB levels in drinkers were higher in GG genotype than in CC or CG genotype (*P *< 0.01 for all). Serum HDL-C levels in drinkers were higher in CG genotype than in CC genotype (*P *< 0.01). Serum TC, TG, HDL-C and ApoA-I levels in CC genotype, TC, HDL-C, ApoA-I levels and the ratio of ApoA-I to ApoB in CG genotype, and TC, TG, LDL-C, ApoA-I and ApoB levels in GG genotype were higher in drinkers than in nondrinkers (*P *< 0.05-0.01). But the ratio of ApoA-I to ApoB in GG genotype was lower in drinkers than in nondrinkers (*P *< 0.01). Multivariate logistic regression analysis showed that the levels of TC, TG and ApoB were correlated with genotype in nondrinkers (*P *< 0.05 for all). The levels of TC, LDL-C and ApoB were associated with genotype in drinkers (*P *< 0.01 for all). Serum lipid parameters were also correlated with age, sex, alcohol consumption, cigarette smoking, blood pressure, body weight, and body mass index in both groups.

**Conclusions:**

This study suggests that the ApoC-III 3238CG heterozygotes benefited more from alcohol consumption than CC and GG homozygotes in increasing serum levels of HDL-C, ApoA-I, and the ratio of ApoA-I to ApoB, and lowering serum levels of TC and TG.

## Introduction

Coronary artery disease (CAD) is the most common cause of death in industrialized countries with evidence that high plasma or serum triglyceride (TG) concentration is an independent risk factor [1-5]. It is well known that plasma TG concentration is modulated by both environmental and genetic factors [6]. Numerous studies have evaluated the influence of alcohol intake, an index of lifestyle, on plasma lipid and lipoprotein concentrations. Alcohol consumption can promote lipogenesis [7] and accordingly increase serum TG levels [8,9]. Alcohol in doses > 30 g/day in both sexes can augment the TG level. It has been found that the alcohol intake of 60 g/day increases the TG level by about 0.19 mg/dl per 1 gram of alcohol consumed [10].

Plasma apolipoprotein (Apo) C-III is a major component of TG-rich lipoproteins (chylomicrons and very low density lipoprotein) and a minor component of high density lipoprotein. The mature 79-amino-acid ApoC-III protein is synthesized predominantly in the liver but also to a lesser extent in the intestine. *In vitro *studies have indicated that ApoC-III is a noncompetitive inhibitor of lipoprotein lipase, thereby suggesting an important role in the catabolism of TG-rich lipoproteins [11]. Plasma ApoC-III concentrations were positively correlated with plasma TG levels, both in the normal population as well as in hypertriglyceridemic patients [12] or in transgenic animals [13]. ApoC-III gene has been mapped to chromosome 11q23.3 [14] and is flanked by the genes for ApoA-I and ApoA-IV in a 15-kb gene cluster [15]. Several polymorphic sites have been detected within and around the ApoC-III gene. The most extensively studied is the *Sst*I polymorphism, due to a C→G substitution at nucleotide 3238, in the 3' untranslated region of the gene. Numerous studies have found an association between the presence of a polymorphic *Sst*I site in the untranslated region of the ApoC-III gene with raised ApoC-III and TG concentrations [16-53] and with an increased risk of CAD [53-62]. However, little is known about the interactions of the ApoC-III gene polymorphism and alcohol consumption on serum lipid concentrations. Therefore, the aim of the present study was to determine the interactions of the ApoC-III 3238C>G (rs5128) polymorphism and alcohol consumption on serum lipid levels.

## Materials and methods

### Study subjects

A total of 1030 unrelated subjects who reside in 16 villages in Napo County, Guangxi Zhuang Autonomous Region, People's Republic of China were randomly selected from our previous stratified randomized cluster samples [63]. The age of the subjects ranged from 15 to 89 years, with an average age of 43.30 ± 17.69 years. There were 516 nondrinkers and 514 drinkers. All of the subjects were peasants. The study subjects were essentially healthy and had no evidence of diseases related to atherosclerosis, CAD and diabetes. None of them had been treated with β-adrenergic blocking agents and lipid-lowering drugs such as statins or fibrates. The present study was approved by the Ethics Committee of the First Affiliated Hospital, Guangxi Medical University. Informed consent was obtained from all subjects after they received a full explanation of the study.

### Epidemiological survey

The survey was carried out using internationally standardized methods, following a common protocol. Information on demographics, socioeconomic status, and lifestyle was collected with standardized questionnaires. Smoking status was categorized into groups of cigarettes per day: <20 and ≥20. Alcohol consumption was categorized into groups of grams of alcohol per day: ≤25 and >25. The physical examination included blood pressure, body height, and body weight, and body mass index (BMI) was calculated as weight (kg) divided by height (m) squared. Sitting blood pressure was measured three times with use of a mercury sphygmomanometer after the subjects had a 5-minute rest, and the average of the three measurements was used in statistical analysis. Systolic blood pressure was determined by the first Korotkoff sound, and diastolic blood pressure by the fifth Korotkoff sound.

### Biochemical analysis

Venous blood samples (8 ml) were drawn from a forearm vein of every subject after venous occlusion for a few seconds in a sitting position, after an overnight fast of 12 h and abstention from alcohol use for at least 12 h. A part of the sample (3 ml) was collected into glass tubes and allowed to clot at ambient temperature, and used to determine serum lipid levels, and another part of the sample (5 ml) was transferred into tubes with anticoagulate solution (4.80 g/L citric acid, 14.70 g/L glucose, and 13.20 g/L tri-sodium citrate) and used to extract deoxyribonucleic acid (DNA). Immediately following clotting serum was separated by centrifugation for 15 minutes at 3000 rpm. The levels of serum total cholesterol (TC), TG, high-density lipoprotein cholesterol (HDL-C), and low-density lipoprotein cholesterol (LDL-C) in samples were determined by enzymatic methods with commercially available kits, Tcho-1, TG-LH (RANDOX Laboratories Ltd., Ardmore, Diamond Road, Crumlin Co. Antrim, United Kingdom, BT29 4QY), Cholestest N HDL, and Cholestest LDL (Daiichi Pure Chemicals Co., Ltd., Tokyo, Japan), respectively. Serum ApoA-I and ApoB levels were assessed by the immunoturbidimetric immunoassay using a commercial kit (RANDOX Laboratories Ltd.). All determinations were performed with an autoanalyzer (Type 7170A; Hitachi Ltd., Tokyo, Japan) in the Clinical Science Experiment Center of the First Affiliated Hospital, Guangxi Medical University.

### DNA amplification and genotyping

Total genomic DNA was isolated from peripheral blood leukocytes using the phenol-chloroform method. The extracted DNA was stored at 4°C until analysis. Genotyping of the ApoC-III 3238C>G was performed by polymerase chain reaction and restriction fragment length polymorphism (PCR-RFLP) according to the previous reports [19]. The sequence of the forward and backward primers used was 5'-CACTAGCCCAGAGAGAGGAGTGCC-3' and 5'-CTGAGCCCAGCCGCACACTAA-3' (Sangon, Shanghai, China). Each reaction system of a total volume of 25 μL, comprised 0.2 μg of genomic DNA; 1.0 μL of each primer (10 pmol/μl); 2.5 μL of 10 × buffer solution; 1.5 μL of MgCl_2 _(25 mmol/L); 2.0 μL of dNTP (2.5 mmol/L); and 1.5 U of Taq polymerase (Takara). For the amplification, initial denaturation at 94°C for 5 minutes was followed by 30 cycles of denaturation at 94°C for 30 s, annealing at 61°C for 30 s, and extension at 72°C for 45 s, with final extension at 72°C for 4 min. Each restriction enzyme reaction was performed with 8 μL of amplified DNA; 2 μL of 10 × buffer solution; and 0.2 U *Sst*I restriction ezyme in a total volume of 25 μL digested at 64°C for 4 h. The digestive products were separated by electrophoresis on 2% sepharose gel for 60 min. The length of each digested DNA fragment was determined by comparing migration of a sample with that of standard DNA marker. Stained with ethidium bromide, the gel was visualized under ultraviolet light and photographed. Genotypes were scored by an experienced reader blinded to epidemiological and lipid results.

### DNA sequencing

Six samples (CC, CG and GG genotypes in two, respectively) detected by the PCR-RFLP were also confirmed by direct sequencing. The PCR product was purified by low melting point gel electrophoresis and phenol extraction, and then the DNA sequence were analyzed by using an ABI Prism 3100 (Applied Biosyatems) in Shanghai Sangon Biological Engineering Technology & Services Co., Ltd., People's Republic of China.

### Diagnostic criteria

The normal values of serum TC, TG, HDL-C, LDL-C, ApoA-I, and ApoB in our Clinical Science Experiment Center were 3.10-5.17, 0.56-1.70, 1.04-1.81, 1.70-3.37 mmol/L, 1.20-1.60, and 0.63-1.14 g/L; respectively [63]. Hypertension was diagnosed according to the criteria of 1999 The World Health Organization-International Society of Hypertension Guidelines for the management of hypertension [64]. The diagnostic criteria of overweight and obesity were according to the Cooperative Meta-analysis Group of China Obesity Task Force. Normal weight, overweight and obesity were defined as a BMI <24, 24-28, and >28 kg/m^2^, respectively [63,64].

### Statistical analysis

Epidemiological data were recorded on a pre-designed form and managed with Excel software. The quantitative variables were presented as mean ± standard deviation (serum TG levels were presented as medians and interquartile ranges). The difference in general characteristics between nondrinkers and drinkers was tested by the Student's unpaired *t *test. The allele frequencies of the ApoC-III 3238C>G were determined by gene counting. A chi-square analysis was used to evaluate the allelic and genotypic frequencies that were calculated from the observed genotypic counts and to assess Hardy-Weinberg expectations. Interaction between the ApoC-III 3238C>G genotype and alcohol consumption was assessed by using a cross-product term between genotypes and the aforementioned factor. Statistical significance was evaluated with analysis of covariance (ANCOVA). The co-variables include sex, age, BMI, hypertension, and cigarette smoking. In order to evaluate the association of serum lipid parameters with several environmental factors and genotypes, unconditional logistic regression analysis was also performed in the combined population, nondrinkers, and drinkers; respectively. The statistical analyses were performed with the statistical software package SPSS 13.0 (SPSS Inc., Chicago, Illinois). A *P *value of less than 0.05 was considered statistically significant.

## Results

### General characteristics between nondrinkers and drinkers

Table 1 gives the general characteristics between the nondrinkers and drinkers. The ratio of male to female, the mean age, the levels of systolic blood pressure, diastolic blood pressure and pulse pressure, and the percentages of subjects who smoked cigarettes were higher in drinkers than in nondrinkers (*P *< 0.05-0.001). There was no significant difference in the BMI between the two groups (*P *> 0.05).

**Table 1:** Comparison of the general characteristics and serum lipid levels between the nondrinkers and drinkers

Parameter	Nondrinker (n = 516)	Drinker (n = 514)	*t *(χ^2^)	*P*
Male/female	186/330	306/208	56.930	0.000
Age (years)	41.26 ± 19.53	45.35 ± 15.38	-3.733	0.000
Body mass index (kg/m^2^)	21.66 ± 2.71	21.95 ± 2.39	-1.821	0.069
Systolic blood pressure (mmHg)	120.03 ± 16.15	125.60 ± 15.63	-5.624	0.000
Diastolic blood pressure (mmHg)	74.13 ± 9.82	77.79 ± 9.78	-5.993	0.000
Pulse pressure (mmHg)	45.95 ± 11.89	47.83 ± 12.01	-2.525	0.012
Cigarette smoking [n(%)]				
Nonsmoker	414(80.2)	292(56.8)		
<20 cigarettes/day	62(12.0)	106(20.6)		
≥20 cigarettes/day	40(7.8)	116(22.6)	69.628	0.000
Alcohol consumption [n(%)]				
Nondrinker	516(100.0)	-		
<25 g/day	-	396(77.0)		
≥25 g/day	-	118(23.0)		
Total cholesterol (mmol/L)	4.52 ± 0.99	4.65 ± 0.95	-2.150	0.032
Triglyceride (mmol/L)^a^	0.97 ± 0.57	1.00 ± 0.61	-2.488	0.013
HDL-C (mmol/L)	1.98 ± 0.45	2.14 ± 0.49	-5.458	0.000
LDL-C (mmol/L)	2.40 ± 0.70	2.41 ± 0.70	-0.229	0.819
Apolipoprotein (Apo) A-I (g/L)	1.40 ± 0.16	1.48 ± 0.13	-8.805	0.000
ApoB (g/L)	0.89 ± 0.22	0.92 ± 0.20	-2.290	0.022
ApoA-I/ApoB	1.68 ± 0.57	1.69 ± 0.46	-0.310	0.757

HDL-C, high-density lipoprotein cholesterol; LDL-C, low-density lipoprotein cholesterol. ^a^The value of TG was presented as median (interquartile range). The difference between the two ethnic groups was determined by the Wilcoxon-Mann-Whitney test.

### Serum lipid levels between nondrinkers and drinkers

The levels of TC, TG, HDL-C, ApoA-I and ApoB were higher in drinkers than in nondrinkers (*P *< 0.05-0.001). There were no significant differences in the levels of LDL-C and the ratio of ApoA-I to ApoB between the two groups (*P *> 0.05 for each).

### Results of electrophoresis and genotyping

**Figure 1:**
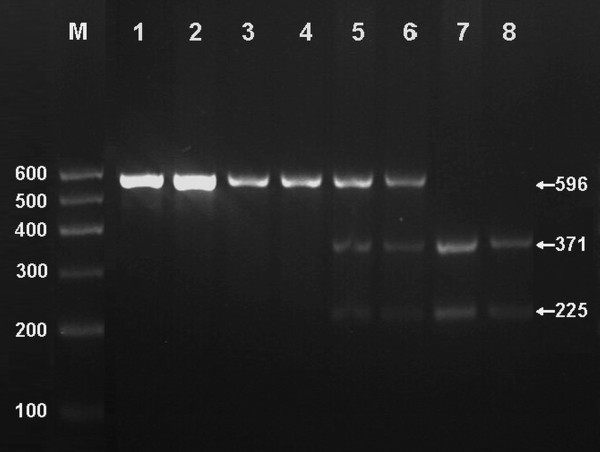
**Genotyping of PCR products of the samples**. Lane M, 100 bp Marker Ladder; Lanes 1 and 2, the PCR products of the samples (596 bp); Lane 3 and 4, CC genotype (596 bp); Lanes 5 and 6, CG genotype (596 bp, 371 bp and 225 bp); and lanes 7 and 8, GG genotype (371 bp and 225 bp).

After the genomic DNA of the samples was amplified by PCR and imaged by 2% agarose gel electrophoresis, the purpose gene of 596 bp nucleotide sequences could be seen in the samples (Figure 1). The genotypes identified were named according to the presence or absence of the enzyme restriction sites, when a C to G transversion at nucleotide position 3238 of the ApoC-III gene. The presence of the cutting site indicates the 3238G allele, while its absence indicates the 3238C allele. Thus, the GG genotype is homozygote for the presence of the site (bands at 371 bp and 225 bp), CG genotype is heterozygote for the presence and absence of the site (bands at 596 bp, 371 bp and 225 bp), and CC genotype is homozygote for the absence of the site (band 596 bp; Figure 1). The genotype distribution was consistent with the Hardy-Weinberg equilibrium.

### Results of sequencing

**Figure 2:**
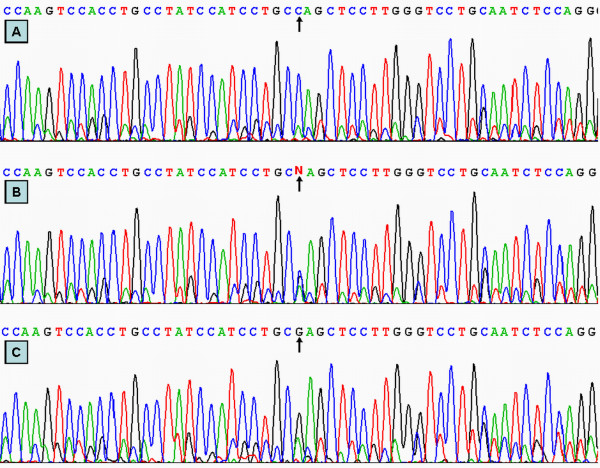
**A part of the nucleotide sequence of the ApoC-III 3238C>G**. (A) CC genotype; (B) CG genotype; (C) GG genotype.

The results shown as CC, CG and GG genotypes by PCR-RFLP, CC, CG and GG genotypes were also confirmed by sequencing (Figure 2).

### Genotypic and allelic frequencies

The genotypic and allelic frequencies of the ApoC-III 3238C>G are shown in Table 2. The frequencies of CC, CG and GG genotypes were 45.7%, 43.0% and 11.3% in nondrinkers, and 45.2%, 45.5% and 9.3% in drinkers (*P *> 0.05); respectively. The frequencies of C and G alleles were 67.2% and 32.8% in nondrinkers, and 67.9% and 32.1% in drinkers (*P *> 0.05); respectively.

**Table 2:** Genotypic and allelic frequencies between the nondrinkers and drinkers [n (%)]

Group	*n*	Genotype			Allele	
			
		CC	CG	GG	C	G
Nondrinker	516	236(45.7)	222(43.0)	58(11.3)	694(67.2)	338(32.8)
Drinker	514	232(45.2)	234(45.5)	48(9.3)	698(67.9)	330(32.1)
χ^2^	-	1.290			0.010	
*P*	-	0.525			0.752	

### Genotypes and serum lipid levels

As shown in Table 3, the levels of TG in nondrinkers were higher in CG genotype than in CC genotype (*P *< 0.01), and the ratio of ApoA-I to ApoB in nondrinkers was higher in GG genotype than in CG genotype (*P *< 0.05).

**Table 3:** Comparison of serum lipid levels among the genotypes and between the nondrinkers and drinkers

Group	Genotype	n	TC (mmol/L)	TG (mmol/L)	HDL-C (mmol/L)	LDL-C (mmol/L)	ApoA-I (g/L)	ApoB (g/L)	ApoA-I/ ApoB
Nondrinker	CC	236	4.49 ± 0.88	0.91 ± 0.57	1.97 ± 0.42	2.41 ± 0.69	1.40 ± 0.13	0.89 ± 0.21	1.70 ± 0.64
	CG	222	4.53 ± 1.11	1.01 ± 0.59^b^	1.98 ± 0.48	2.40 ± 0.68	1.40 ± 0.18	0.91 ± 0.22	1.62 ± 0.41
	GG	58	4.57 ± 1.03	0.93 ± 0.60	2.01 ± 0.43	2.40 ± 0.83	1.42 ± 0.12	0.86 ± 0.28	1.84 ± 0.75^c^
*F*	-	-	0.186	12.517	0.187	0.013	0.445	1.275	3.685
*P*	-	-	0.830	0.002	0.829	0.987	0.641	0.280	0.026
Drinker	CC	232	4.72 ± 0.90**	0.97 ± 0.63**	2.07 ± 0.45*	2.38 ± 0.71	1.47 ± 0.14**	0.91 ± 0.20	1.70 ± 0.46
	CG	234	4.75 ± 0.86*	0.95 ± 0.54	2.23 ± 0.53^b^**	2.37 ± 0.66	1.49 ± 0.13**	0.91 ± 0.20	1.73 ± 0.47**
	GG	48	5.29 ± 0.91^bd^**	1.28 ± 0.93^bd^**	2.12 ± 0.49	2.77 ± 0.72^bd^*	1.49 ± 0.14**	1.06 ± 0.18^bd^**	1.44 ± 0.30^bd^**
*F*	-	-	8.662	18.352	6.253	7.173	1.382	12.457	8.300
*P*	-	-	0.000	0.000	0.002	0.000	0.252	0.000	0.000

TC, total cholesterol; TG, triglyceride; HDL-C, high-density lipoprotein cholesterol; LDL-C, low-density lipoprotein cholesterol; ApoA-I, apolipoprotein A-I; ApoB, apolipoprotein B. The value of TG was presented as median (interquartile range). The difference among the genotypes was determined by the Kruskal-Wallis test. ^a^*P *< 0.05 and ^b^*P *< 0.01 in comparison with CC genotype of the same subgroup;^c^*P *< 0.05 and ^d^*P *< 0.01 in comparison with CG genotype of the same subgroup;*P < 0.05 and **P < 0.01 in comparison with the same subgroup of the nondrinkers.

The levels of TC, TG, LDL-C and ApoB in drinkers were higher in GG genotype than in CC or CG genotype (*P *< 0.01 for all). The levels of HDL-C in drinkers were higher in CG genotype than in CC genotype (*P *< 0.01). The ratio of ApoA-I to ApoB in drinkers was lower in GG genotype than in CC or CG genotype (*P *< 0.01 for each).

Serum TC, TG, HDL-C and ApoA-I levels in CC genotype, TC, HDL-C, ApoA-I levels and the ratio of ApoA-I to ApoB in CG genotype, and TC, TG, LDL-C, ApoA-I and ApoB levels in GG genotype were higher in drinkers than in nondrinkers (*P *< 0.05-0.01). But the ratio of ApoA-I to ApoB in GG genotype was lower in drinkers than in nondrinkers (*P *< 0.01).

### Correlation between genotype and serum lipid parameters

Multivariate logistic regression analysis showed that the levels of TC, TG LDL-C and ApoA-I were correlated with genotype in the combined population (*P *< 0.05-0.01). The levels of TC, TG and ApoB were correlated with genotype in nondrinkers (*P *< 0.05 for all). The levels of TC, LDL-C and ApoB were associated with genotype in drinkers (*P *< 0.01 for all). Serum lipid parameters were also correlated with age, sex, alcohol consumption, cigarette smoking, blood pressure, body weight, and BMI in both nondrinkers and drinkers (Table 4).

**Table 4:** Correlative factors for the serum lipid parameters between the nondrinkers and drinkers

Lipid parameter	Risk factor	Odds ratio	χ^2^	*P*	95% CI
Nondrinkers plus drinkers					
TC	Age	1.030	45.053	0.000	1.021-1.039
	Body mass index	1.144	22.000	0.000	1.081-1.210
	ApoC-III 3238C>G genotype	1.324	6.542	0.011	1.068-1.642
TG	Body mass index	1.223	30.735	0.000	1.139-1.314
	Pulse pressure	1.018	5.255	0.022	1.003-1.034
	Alcohol consumption	1.744	14.249	0.000	1.307-2.329
	Cigarette smoking	0.614	4.056	0.044	0.382-0.987
	ApoC-III 3238C>G genotype	1.517	8.448	0.004	1.145-2.009
LDL-C	Age	1.030	14.365	0.000	1.014-1.046
	Sex	1.898	5.549	0.018	1.114-3.236
	Body weight	1.107	43.419	0.000	1.074-1.141
	Alcohol consumption	0.704	4.248	0.039	0.504-0.983
	ApoC-III 3238C>G genotype	1.709	9.600	0.002	1.218-2.400
ApoA-I	Age	0.745	12.294	0.000	0.632-0.878
	Sex	0.414	9.812	0.002	0.238-0.719
	Pulse pressure	0.974	4.009	0.045	0.949-0.999
	Alcohol consumption	0.327	12.749	0.000	0.177-0.604
ApoB	Age	1.036	38.386	0.000	1.025-1.048
	Body weight	1.086	45.102	0.000	1.060-1.113
	Alcohol consumption	3.154	7.351	0.007	1.375-7.234
	ApoC-III 3238C>G genotype	1.678	15.923	0.000	1.301-2.163
	Sex	1.678	3.923	0.048	1.004-2.219
Nondrinkers					
TC	Age	1.142	13.511	0.000	1.064-1.225
	Body mass index	1.175	14.633	0.000	1.082-1.277
	ApoC-III 3238C>G genotype	1.324	6.542	0.011	1.068-1.642
	Cigarette smoking	0.639	4.668	0.031	0.425-0.959
TG	Body mass index	1.163	7.954	0.005	1.047-1.292
	Age	1.314	9.279	0.002	1.102-1.566
	Cigarette smoking	0.280	5.302	0.021	0.095-0.827
	ApoC-III 3238C>G genotype	2.200	12.048	0.044	1.410-3.434
LDL-C	Age	1.039	13.847	0.000	1.018-1.060
	Body weight	1.131	34.897	0.000	1.086-1.178
	Sex	3.379	9.542	0.002	1.561-7.317
ApoA-I	Body weight	1.061	4.339	0.037	1.004-1.122
	Body mass index	0.671	14.044	0.000	0.545-0.827
	Age	0.733	11.783	0.001	0.614-0.875
	Cigarette smoking	1.667	4.219	0.040	1.024-2.713
ApoB	Age	1.143	11.084	0.001	1.057-1.237
	Body weight	1.084	25.440	0.000	1.051-1.119
	ApoC-III 3238C>G genotype	1.574	6.223	0.013	1.102-2.248
Drinkers					
TC	Age	1.326	15.892	0.000	1.154-1.523
	Body weight	1.052	12.961	0.000	1.023-1.082
	ApoC-III 3238C>G genotype	1.874	16.157	0.000	1.380-2.545
TG	Alcohol consumption	0.674	10.599	0.001	0.532-0.855
	Body mass index	1.270	23.776	0.000	1.154-1.399
LDL-C	Height	1.063	7.372	0.007	1.017-1.112
	ApoC-III 3238C>G genotype	2.319	10.577	0.001	1.397-3.849
ApoA-I	Body weight	1.078	6.384	0.037	1.017-1.143
	Systolic blood pressure	0.942	7.787	0.005	0.903-0.982
ApoB	Age	1.040	17.141	0.000	1.021-1.060
	Body weight	1.084	22.706	0.000	1.049-1.121
	Pulse pressure	0.974	5.365	0.021	0.952-0.996
	Alcohol consumption	0.360	13.214	0.000	0.207-0.624
	ApoC-III 3238C>G genotype	1.863	11.039	0.001	1.291-2.689

TC, total cholesterol; TG, triglyceride; LDL-C, low-density lipoprotein cholesterol; ApoA-I, apolipoprotein A-I; ApoB, apolipoprotein B; CI, confidence interval

## Discussion

The results of the present study show that the levels of TC, TG, HDL-C, ApoA-I and ApoB were higher in drinkers than in nondrinkers. There was no significant difference in the levels of LDL-C and the ratio of ApoA-I to ApoB between the two groups. These findings are consistent with those of several previous studies. A moderate intake of alcohol is associated with protection against CAD, probably due in part to a dose-dependent increase in HDL-C [65,66]. According to Rimma *et al. *[67], a daily dose of 30 g alcohol results in an average HDL level rise of 3.99 mg/dl, and an ApoA-I level rise of 8.82 mg/dl. Alcohol also causes an increase of TG lipase activity and a decrease of the HDL removal from the circulation [68]. A decrease in LDL-C with increased alcohol intake has also been reported in some studies, but this effect is less consistent and probably depends on the combination of one or more unmeasured factors [68].

The present study shows that there was no significant difference in the allelic and genotypic frequencies of the ApoC-III 3238C>G between the nondrinkers and drinkers. The frequency of G allele was 32.8% in nondrinkers, and 32.1% in drinkers, which is quite similar to the results in Taiwanese (0.30-0.43) [46,69], Japanese (0.25-0.48) [70], and Indians (0.36) [27], but is higher than those reported for Caucasians in whom the G allele frequency was 0.00-0.11 [27,55]. These results suggest that there exists significant racial variation of allele frequencies in this locus.

The relationship between the ApoC-III 3238C>G polymorphism and plasma or serum lipid levels in humans has been evaluated in a large number of studies. However, previous findings on the association of this polymorphism with the changes in plasma lipid levels are inconsistent [71-73]. Previous cohort studies, as well as case-control and familial studies have shown significant association between the rare allele of the polymorphic *Sst*I site (3238G) and higher plasma TG levels [16-53] and CAD [53-62]. This association has been reported in studies carried out with Caucasians, Chinese, Mayans, Japanese (living in Japan or living in Southern Brazil), Koreans, Arabs, and Asian Indians [27,49,55,69,70]. However, several reports failed to find a significant genetic effect on TG concentrations [71-73]. In a previous work, Kee and coworkers found no association between variability at the *Sst*I ApoC-III gene site (in the 3%-noncoding region) and lipid, lipoproteins and complex lipoprotein particles in a sample of men from northern France [72]. They thought that the *Sst*I polymorphism is not major contributors to the risk of dyslipidemia in the population of northern France. Results from the current study are consistent with many studies cited above which reported associations of ApoC-III gene polymorphism with altered lipid metabolism. The levels of TG in nondrinkers were higher in CG genotype than in CC genotype, and the ratio of ApoA-I to ApoB in nondrinkers was higher in GG genotype than in CG genotype. The levels of TC, TG, LDL-C and ApoB in drinkers were higher in GG genotype than in CC or CG genotype. The levels of HDL-C in drinkers were higher in CG genotype than in CC genotype. The ratio of ApoA-I to ApoB in drinkers was lower in GG genotype than in CC or CG genotype.

The interactions of the ApoC-III 3238C>G polymorphism and alcohol consumption on serum lipid levels are not well known. In the present study, we showed that serum TC, TG, HDL-C and ApoA-I levels in CC genotype, TC, HDL-C, ApoA-I levels and the ratio of ApoA-I to ApoB in CG genotype, and TC, TG, LDL-C, ApoA-I and ApoB levels in GG genotype were higher in drinkers than in nondrinkers. But the ratio of ApoA-I to ApoB in GG genotype was lower in drinkers than in nondrinkers. The levels of TG were correlated with genotype in nondrinkers, whereas the levels of TG were positively associated with alcohol consumption in drinkers. These findings suggest that the ApoC-III 3238CG heterozygotes benefited more from alcohol consumption than CC and GG homozygotes in increasing serum levels of HDL-C, ApoA-I, and the ratio of ApoA-I to ApoB, and lowering serum levels of TC and TG. The effect of different kinds of wine on the lipid profiles is not well known. In a previous study, Ruidavets *et al. *[74] found that wine was positively associated with HDL-C. Beer was positively associated with HDL-C in men and with TGs in men and women. When taking drinking patterns into account, wine drinkers had higher HDL-C levels than non-wine drinkers. In another study, Choudhury *et al. *[75] also showed serum TGs levels were significantly lower in those who drank beer. Thus, we hypothesize that the interactions between the ApoC-III 3238C>G polymorphism and different kinds of alcoholic beverage on serum lipid levels may be different.

## Conclusion

The results of the present study show that there was no significant difference in genotypic and allelic frequencies of the ApoC-III 3238C>G polymorphism between the nondrinkers and drinkers. But the interactions of the ApoC-III 3238C>G polymorphism and alcohol consumption on serum lipid levels are different among the three genotypes. The ApoC-III 3238CG heterozygotes benefited more from alcohol consumption than CC and GG homozygotes in increasing serum levels of HDL-C, ApoA-I, and the ratio of ApoA-I to ApoB, and lowering serum levels of TC and TG.

## Competing interests

The authors declare that they have no competing interests.

## Authors' contributions

YR and LY conceived the study, participated in the design, carried out the epidemiological survey, collected the samples, performed the statistical analyses, and drafted the manuscript; LM, LK, LX, ZL and LW carried out the biochemical analysis; WJ, YD and LW carried out the epidemiological survey, collected the samples, and helped to carry out the genotyping. All authors read and approved the final manuscript.
